# Psychiatric manifestations in Neuronal ceroid lipofuscinoses

**DOI:** 10.1186/s13023-026-04416-0

**Published:** 2026-06-15

**Authors:** Stefania Della Vecchia, Alessandro Simonati, Maria Marchese, Stefano Berloffa, Alfried Kohlschütter, Filippo Maria Santorelli

**Affiliations:** 1 IRCCS Stella Maris Foundation, via dei Giacinti 2, 56128 Calambrone, Pisa Italy; 2 Department of Neurosciences, Psychology, Drug Research and Child Health (NEUROFARBA), University of Florence, Viale Pieraccini, 6, 50139 Florence, Italy; 3 Department of Surgery, Dentistry, Paediatrics and Gynaecology, University of Verona, 37134 Verona, Italy; 4 Department of Pediatrics, University Medical Center Eppendorf, 20246 Hamburg, Germany; 5 Molecular Medicine, IRCCS Fondazione Stella Maris – via dei Giacinti, 56128 Pisa, Italy

**Keywords:** NCL, Psychiatric symptoms, Psychopathology, Degenerative brain disease in children and young adults

## Abstract

**Background:**

Neuronal ceroid lipofuscinoses (NCLs) are rare genetic neurodegenerative disorders characterized by progressive cognitive, motor, and visual decline. The transition from supportive care to emerging disease-modifying therapies has reshaped the clinical landscape of NCLs and highlighted the urgent need for sensitive and clinically meaningful outcome measures beyond traditional motor and survival endpoints. Psychiatric manifestations, which profoundly affect daily functioning and quality of life, represent a potentially valuable yet underexplored domain.

**Methods:**

We conducted a systematic review following PRISMA guidelines to synthesize current evidence on the prevalence, clinical spectrum, developmental trajectories, assessment strategies, and management of psychiatric manifestations across NCL subtypes.

**Results:**

Psychiatric symptoms are highly prevalent across NCL forms and encompass a broad clinical spectrum, including early emotional and behavioral dysregulation, neurodevelopmental-like features, anxiety and depressive symptoms, obsessive–compulsive phenomena, and psychotic-spectrum manifestations. Their expression varies according to age and disease stage and reflects a multifactorial interplay between neurodegeneration, disrupted neurodevelopment, autonomic and sensory dysfunction, cognitive decline, genetic modifiers, and environmental factors. Despite their clinical relevance, management remains largely empirical and palliative, relying on psychotropic medications and supportive interventions with limited evidence and frequent adverse effects. Furthermore, psychiatric assessment tools are inconsistently applied, predominantly in CLN3 disease, and terminology remains heterogeneous and non-standardized, hindering cross-study comparability and robust phenotypic characterization.

**Conclusions:**

Psychiatric manifestations represent a core and developmentally modulated component of NCLs. The lack of standardized language and validated, age-appropriate assessment instruments constitute a major barrier to clinical care and research. Systematic phenotyping and harmonized measurement strategies are urgently needed to improve management and to integrate psychiatric outcomes into future natural history studies and therapeutic trials.

**Supplementary Information:**

The online version contains supplementary material available at https://doi.org/10.1186/s13023-026-04416-0.

## Introduction

Neuronal ceroid lipofuscinoses (NCLs) are the most common group of pediatric neurodegenerative diseases [1]. Although onset usually occurs in childhood, later-onset forms have also been described [2]. These disorders are caused by genetic mutations (over 13 forms have been identified, CLN1–CLN14) and are characterized by progressive cognitive and motor decline, epilepsy, and vision loss. Approved disease modifying treatments do not exist outside of CLN2 disease. However, in recent years, several novel experimental therapies have emerged, including both gene-based and non–gene-based approaches. These therapies require clinical trials with robust endpoints to properly assess their efficacy, capable of detecting even small yet clinically meaningful changes in quality of life and disease burden.

In this scenario, it has emerged the urgency for outcome measures capable of detecting minimum changes. The traditional scales for the disease with motor and cognitive outcomes may not be sufficient to fully capture the complexity of these conditions. Hence the need to investigate other areas of the disease that have been less characterised to date but which have a significant impact on quality of life. Among them, psychiatric symptoms deserve particular attention as potential outcome measures for future clinical trials capable of reflecting real-life functioning. In adult neurodegenerative disorders neuropsychiatric symptoms are already used as formal outcomes in interventional studies [3, 4]. They are common in clinical practice and have been documented in NCLs since the earliest descriptions [5], but remain poorly characterized from both a phenotypic and therapeutic perspective. They may herald disease onset, accompany neurological progression, and substantially complicate both clinical presentation and daily management. Unanswered questions concern the full spectrum and temporal trajectories of psychiatric manifestations, including potential differences between genotypes and triggers of their expression. As a result, clinicians are confronted with a poorly characterized and clinically challenging domain, both in terms of symptomatology and underlying neurobiology, leading to significant challenges in the care of patients and families.

Based on this, our review aims to summarise evidence on the prevalence, clinical spectrum, and current management of psychiatric manifestations in NCL subtypes, identify critical gaps in this area, and propose recommendations for incorporating standardised psychiatric assessment into clinical care and research of NCLs.

## Materials and methods

**Fig. 1:**
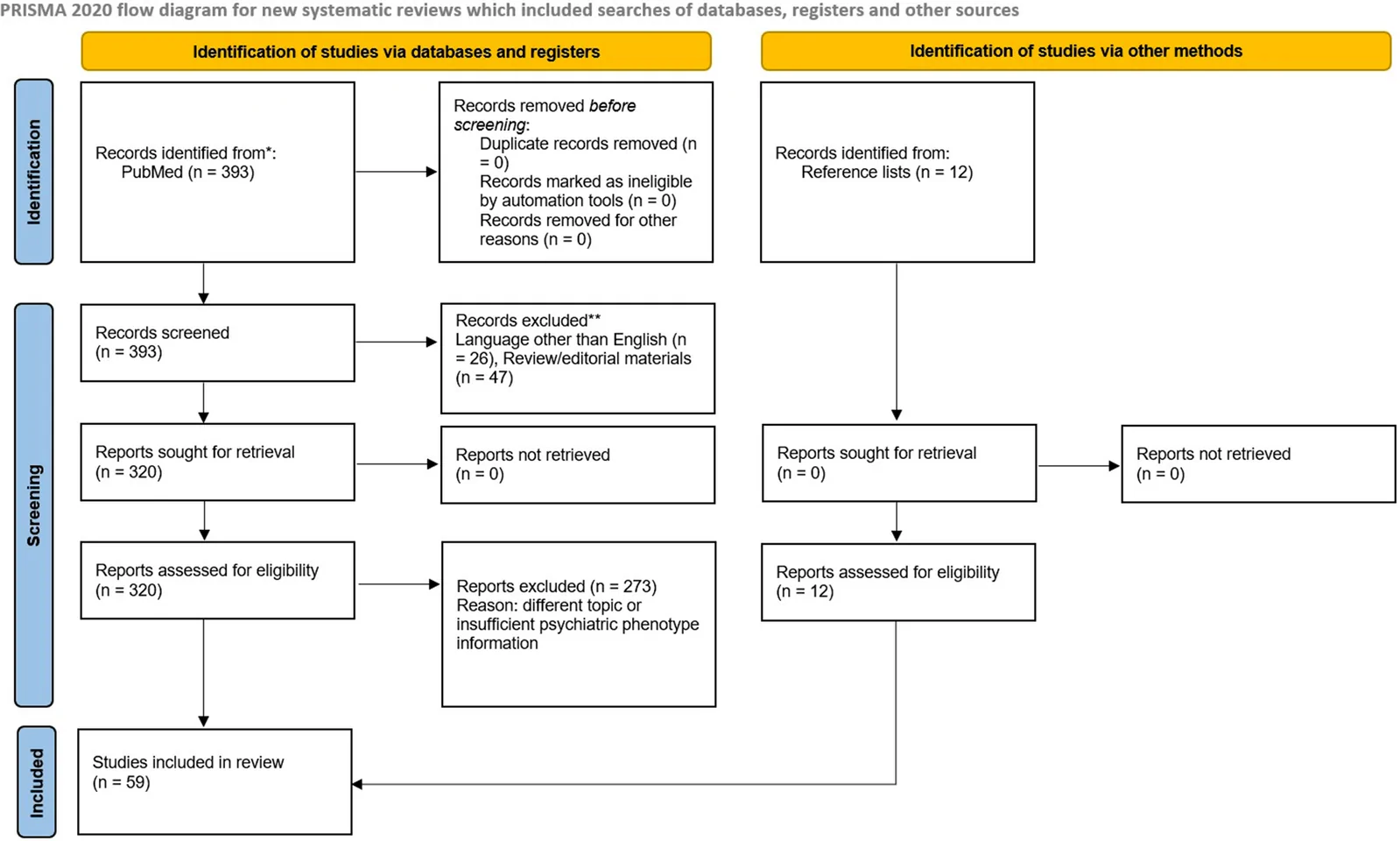
PRISMA flow chart. The diagram illustrates the search and selection of papers for this review

A systematic literature search was conducted in accordance with the Preferred Reporting Items for Systematic Reviews and Meta-Analyses (PRISMA) guidelines [6] using PubMed/MEDLINE from database inception to [3 August 2025] (no date limits) to identify studies reporting psychiatric or behavioral manifestations in individuals with genetically or pathologically confirmed neuronal ceroid lipofuscinosis (any subtype). The search strategy was the following: (“neuronal ceroid lipofuscinos*” OR “Batten disease” OR “CLN1” OR “CLN2” OR “CLN3” OR “CLN4” OR “CLN5” OR “CLN6” OR “CLN7” OR “CLN8” OR “CLN10” OR “CLN11” OR “CLN12” OR “CLN14”) AND (“psychiatric” OR “psychos*” OR “hallucination*” OR “delusion*” OR " schizophrenia*” OR “cataton*” OR “depression” OR “depressive” OR “bipolar disorder” OR “bipolar” OR “mania” OR “mood disorder*” OR “anxiety” OR “psychiatric” OR “psychopathology” OR “behavio*” OR “behaviou*” OR “autism” OR “autistic” OR “ASD” OR “Autism Spectrum Disorders (ASD)” OR “Rett-like” OR “ADHD” OR “hyperactivity” OR “attention deficit” OR “obsessive compulsive” OR “OCD” OR “emotional” OR “aggression” OR “aggressive” OR “irritability” OR “oppositional behavior” OR “oppositional behavior”). Zotero 5.0 reference manager was used to exclude duplicate papers. The full text of all 393 potentially eligible articles and their supplementary information was obtained and independently assessed by two authors (F.M.S. and S.D.V.), as seen in Fig. 1. Disagreements regarding study eligibility were resolved through discussion. Eligibility criteria included peer-reviewed, full-text human studies (cohort, cross-sectional, case–control, case series, and single cases) that provided sufficient detail on psychiatric outcomes or used standardized instruments. We restricted inclusion to English-language full texts; non-English full texts and English abstracts of non-English articles were excluded. We also excluded reviews (narrative/systematic), editorials, commentaries, letters without extractable data, conference abstracts without a corresponding full text, theses/dissertations, book chapters, protocols, and preclinical/animal/in vitro studies. From each included study we extracted design, sample and setting, NCL subtype/genotype, age at evaluation, definitions and timing of psychiatric outcomes, assessment tools, interventions, and key findings. Owing to heterogeneity of designs, measures, and small samples, no meta-analysis was attempted; instead, we performed a qualitative synthesis. The review aimed to map the spectrum and trajectories of psychiatric features, summarize the use of standardized assessments, and outline practical implications for clinical management.

Supplementary Table 1 lists the included 59 studies included in this review.

## Results

### Overview of published studies on psychiatric manifestations in NCLs

**Fig. 2:**
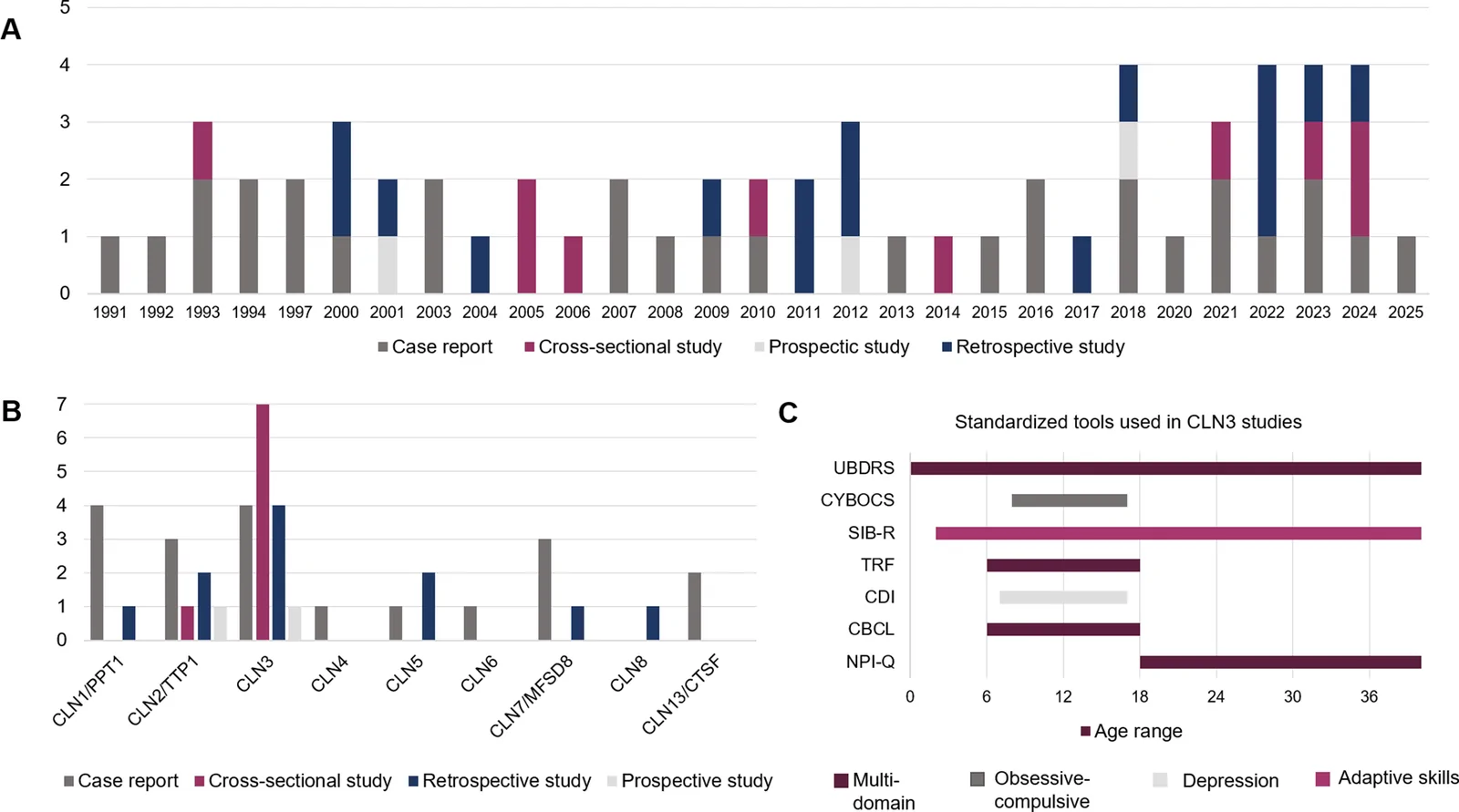
Overview of the published literature on psychiatric manifestations in NCLs. (**A**) Temporal distribution and study design of publications addressing psychiatric symptoms in NCLs since the discovery of causative genes (period 1991–2025). (**B**) Distribution and type of studies according to NCL genotype. We considered only publications that explicitly report genetic diagnosis. (**C**) Standardized assessment tools used in studies on CLN3 disease, with bars indicating the recommended age range for administration. Colors denote the primary symptom domains assessed. UBDRS: Unified Batten Disease Rating Scale, CYBOCS: Children’s Yale-Brown Obsessive Compulsive Scale, SIB-R: Scales of Independent Behavior – Revised, TRF: Teacher’s Report Form, CDI – Children’s Depression Inventory, CBCL: Child Behavior Checklis, NPI-Q: Neuropsychiatric Inventory – Questionnaire

Research on psychiatric manifestations in NCLs is largely based on small case reports and series and retrospective or cross-sectional cohorts (Fig. 2A). The most investigated form is the juvenile onset CLN3 disease, whereas information on other subtypes (CLN1, CLN2, CLN5, CLN6, CLN7, CLN8) remain poor (Fig. 2B). Psychiatric features are usually described with non-specific terms and without standardized diagnostic criteria. A limited number of studies about CLN3 disease employed validated tools for pediatric population (Fig. 2C) [7–13]. A single adult case employed the Bush–Francis Catatonia Rating Scale to assess catatonia [14]. Supplementary table S2 described in detail the psychiatric tools used in NCL studies. The UBDRS, developed specifically for juvenile NCLs, also incorporates behavioral domains [8] and shows substantial agreement with the CBCL questionnaire [10]. However, most UBDRS-based studies reported behavioral symptoms only as present or absent without a more in-depth definition of the psychiatric clinical picture [15, 16]. More recently, Honingh and colleagues [12] expanded the psychiatric domains identified in CLN3 patients and underscoring the need to update the disease-specific scales. The gap is even more pronounced in preschool-aged children, for whom standardised characterisation is still lacking.

### The psychiatric spectrum of NCLs: from irritability to psychosis

Although not yet systematically characterized, numerous reports and studies on NCLs have described the presence of psychiatric symptoms in this population. This is even more evident in clinical practice, where such manifestations are frequently observed. To summarize the major psychiatric features across different NCL subtypes (Table 1), symptoms were organized according to dimensional and transdiagnostic psychopathological domains, distributed along a continuum of severity and shared across multiple diagnostic categories. Indeed, in most studies, symptoms are not reported according to DSM-5 diagnostic categories.

**Table 1:** Most common psychiatric manifestations in different subtypes of NCLs

Symptoms	Most Common Age Range	NCL subtypes	References
Irritability	Infancy, Childhood	CLN1, CLN2, CLN8, CLN3	[5, 9, 15–18].
Aggressiveness	Childhood	CLN1, CLN2, CLN5, CLN7, CLN3	[5, 9, 16–20]
Oppositional behavior	Childhood	CLN1, CLN8	[5, 19]
Hyperactivity	Infancy, Childhood	CLN1, CLN2, CLN5, CLN8	[5, 16–20]
Attention problems	Childhood	CLN2, CLN5, CLN1, CLN3	[5, 9, 21]
Autistic traits	Infancy, Childhood	CLN1, CLN2, CLN5, CLN7, CLN3	[18, 22–31]
Depressive symptoms	Adolescence, Adulthood	CLN5, CLN3, CLN13	[5, 7, 9, 14, 32, 33]
Anxiety symptoms	Adolescence	CLN1, CLN5, CLN8, CLN3	[7, 32, 34, 35]
Obsessive–compulsive symptoms	Adolescence	CLN2, CLN5, CLN3	[9, 19, 32]
Psychotic-spectrum symptoms	Adolescence, Adulthood	CLN5, CLN2, CLN1, CLN3, ANCL	[5, 7, 9, 14, 19, 36–44]

Early emotional dysregulation and behavioral problems emerge as the most consistent and recurrent findings across NCL subtypes. Irritability, hyperactivity, oppositional behaviors, and aggression are frequently reported and may precede neurological signs such as seizures or visual decline. In CLN1 disease, irritability has been described as an extremely early feature, affecting up to 90% of patients between the age of 1 and 3 years and persisting in 60% of the children beyond age 6–8 [5, 15–18]. Oppositional behavior and aggressive outbursts have also been reported [5, 19]. Similar features are found in CLN2 patients [16, 45, 46], especially in its so called “variant form” where such disturbances may precede in about 16% of the cases [21], other clinical signs [47] including seizures and epilepsy [48]. CLN3 is characterized by a high prevalence of behavioral problems like attention problems and aggressive behavior in early school age [7, 9]: cognitive and behavioral disturbances were the first symptom to alert parents in 19% of patients and the second or third symptom in 29% and 54% of cases, respectively [8]. In CLN5, cohort studies indicate behavioral disturbances such as hyperactivity and aggression in 57–73% of patients [19, 49], with behavioral manifestations occurring at onset in more than one third of cases [19] and seldom dominating the clinical picture [50]. Likewise, nearly half of CLN8 patients exhibited irritability, hyperactivity, disobedience, and inattention during childhood [51]. Collectively, these data suggest that behavioral dysregulation is a core and early feature across several NCLs and, in some subtypes, may represent as an early marker to consider a NCL diagnosis.

A second set of manifestations concerns social and relational problems, which often resemble autistic spectrum traits or syndromic autism. Rett-like phenotypes have been described in CLN1 disease [18, 22, 23, 25] and in classic vLINCL forms such as CLN6 and CLN7 [24, 30, 52]. A wide range of presentations has been observed, including stereotyped hand movements, regression in social and communicative abilities, and psychomotor regression. In a group of CLN7 adolescents, aggressive and unsociable behaviors resembled ASD-like traits were reported. However, a formal ASD diagnosis and assessment were not documented [31]. In CLN2, autistic-like traits, social and communication difficulties, and repetitive behaviors have been described [26, 27]. Patients with CLN3 are often described as socially withdrawn and have difficulty interacting with peers [28, 29, 53]. These reports confirm that autistic traits and relational difficulties are not confined to a single NCL subtype, but rather appear across the disease spectrum, particularly in early-onset forms.

Depression and anxiety symptoms are other important clinical dimensions. In CLN1, CLN2, and CLN5, depressive and anxious symptoms typically emerge alongside disease progression, evolving from childhood irritability and aggression [15–20]. Adult-onset forms also present with mood and anxiety disturbances [54, 55]. CLN3 is the most well-documented subtype. Santavuori et al. [5] described depression as a baseline emotional state, and subsequent studies estimated a relative prevalence ranging from low to about 18%. These features were generally mild and, in some cases, responsive to treatment with citalopram. Anxiety is even more common, with a prevalence as high as 70% in a parental survey [32]. Autonomic dysfunction and paroxysmal sympathetic hyperactivity seem to been linked to worsening anxiety in CLN3 [34, 35].

Obsessive–compulsive symptoms has been described particularly for CLN3, where estimate prevalence ranges between 56% [9] to 65.1% [32]. Obsessions include animals or imaginary characters, fears of contamination, preoccupation with appearance, or intrusive thoughts about anticipated events, while compulsions typically involve hoarding, ordering, or repetitive checking [9]. Importantly, the compulsions observed in CLN3 differ from those in classical OCD in that they are often stereotyped and lack a goal-directed or harm-preventive intent [56]. This perseverance style is distinct from behaviors observed in conditions such as developmental delay, autism, and other psychiatric or neurological disorders [57]. Furthermore, obsessive fears in JNCL have been linked to visual hallucinations, further complicating differential diagnoses [5, 58]. In other subtypes, obsessive-compulsive manifestations have only been anecdotally reported [19].

Finally, psychotic features represent another clinically relevant component of the NCL phenotype. Psychotic symptoms tend to emerge at a later stage in the progression of pediatric forms of NCLs. This pattern has been observed in CLN1 [17, 40, 59], CLN2 [60] and CLN5 [19]. Standardized assessments confirm thought disorder, delusions, and hallucinations in CLN3 [7, 9]. In adult-onset NCLs (ANCL, including Kufs disease), psychosis may occur early on and even represent the first manifestation of the disease [35–43]. The prevalence across juvenile [61] and adult-onset subtypes [36–39, 41–44, 62] has been estimated at approximately 20%, with symptom onset occurring between the ages of 13 and 41. Hallucinations, delusions, and thought disorder are the typical psychotic symptoms reported in these patients [42]. Catatonic motor behavior is occasionally reported as well [36]. However, the terminology used to describe these symptoms is often inconsistent. The precise nature of hallucinations is not always defined, and reports may refer to thought disorders [7], suspiciousness [43], or paranoia [43] and the precise nature of hallucinations is not always defined. Visual hallucinations are frequently reported [15, 37, 40, 63, 64] but auditory hallucinations are also documented [37, 40, 44, 64–66]. In some cases, these symptoms have led to an initial misdiagnosis of schizophrenia [44]. It is important to say that seeing things that are not there (hallucinations) has been described as “release” hallucinations in blind patients with NCL [67]. This happens when sensory loss stops higher brain cells from working together [68]. This lets brain cells’ natural activity be seen as sensory input. This makes it seem like real sensory experiences are happening. To make an accurate diagnosis and provide the right treatment, it is essential to carefully evaluate hallucinations and determine if they are psychotic, epileptic, or related to a release.

### Developmental trajectories of psychiatric symptoms in NCLs

The absence of prospective studies has thus far precluded the establishment of a longitudinal trajectory of psychiatric symptoms in NCL. Nonetheless, developmental trends are reported.

In infantile and late-infantile NCL forms (e.g., CLN1, CLN2, CLN5, CLN7, and CLN8), early psychiatric presentations may include irritability, aggressive and oppositional behaviours, hyperactivity, and autism spectrum–like traits [5, 15–18]. These manifestations often reflect prominent emotional and behavioural dysregulation, which can phenotypically overlap with neurodevelopmental disorders and may co-occur with ADHD- or ASD-like features. Accordingly, some patients are initially referred for, and assessed within, a neurodevelopmental framework. This suggests that the developmental program of brain maturation remains partially active in NCLs but is progressively disrupted by neurodegenerative processes. However, the clinical picture typically evolves over time, with the emergence of neurological “red flags” such as seizures and progressive motor impairment. In addition, language development may be arrested, and progressive language deterioration or broader cognitive regression can become evident.

In juvenile forms, particularly CLN3, early manifestations may include emotional dysregulation, attentional difficulties, hyperactivity, oppositional behaviour, and, in some cases, social withdrawal [7, 9]. Although progressive visual failure represents the hallmark feature of CLN3, behavioural and attentional symptoms may precede a formal ophthalmological diagnosis. Social withdrawal, in particular, may partly reflect early, unrecognized visual impairment rather than a primary psychopathological process. With school entry, difficulties in academic performance are often reported, potentially related to emerging visual deficits and/or early cognitive involvement. As the disease progresses, additional psychiatric manifestations may emerge, including anxiety and depressive symptoms, repetitive behaviours and obsessive features, as well as psychotic symptoms—sometimes subthreshold—such as hallucinations and delusions [7, 32, 34, 35]. In adult-onset forms, psychiatric symptoms may constitute the presenting feature, preceding overt neurological signs in a subset of patients [5, 7, 9, 19, 36–44].

**Fig. 3:**
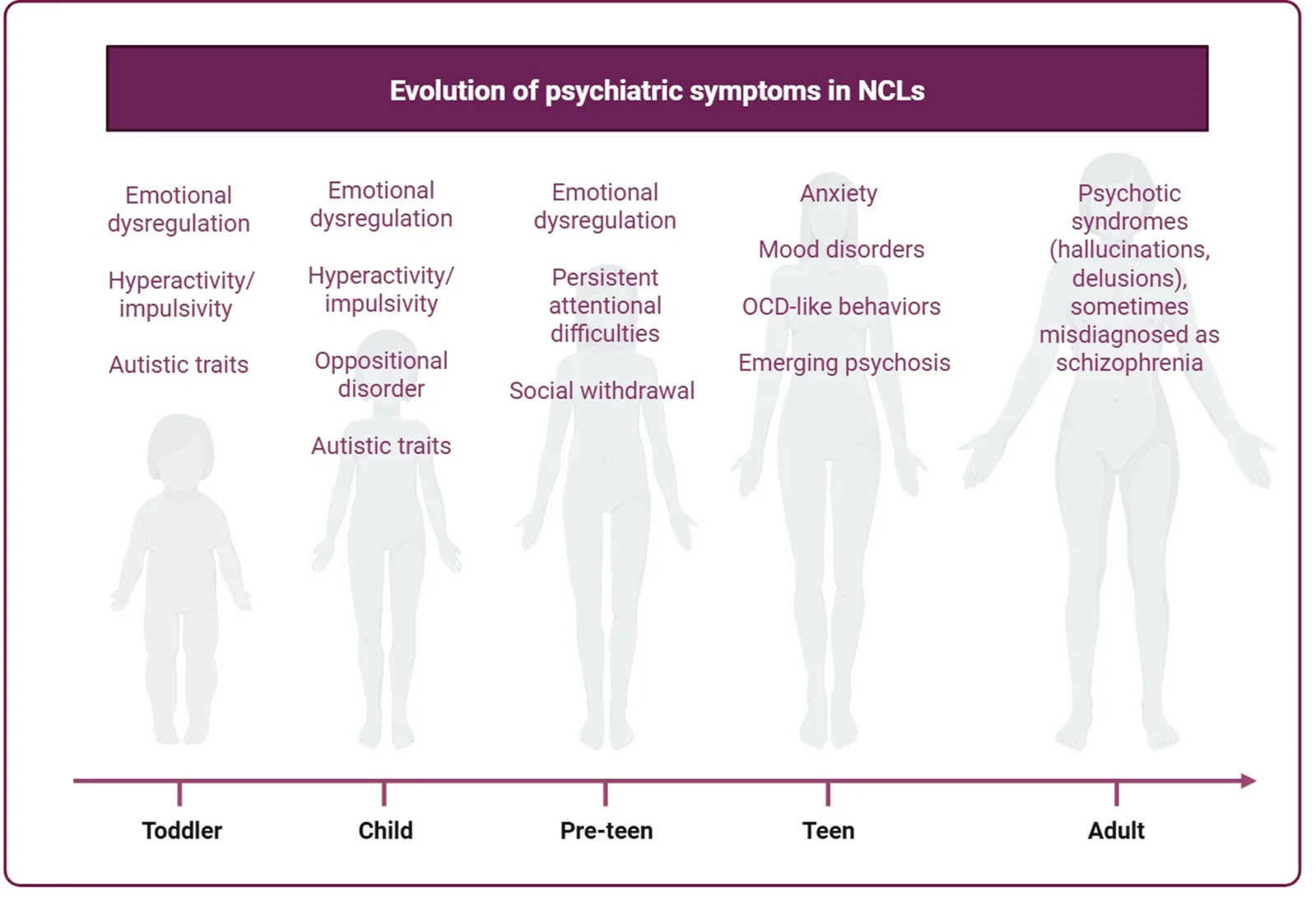
Main psychiatric phenotypes across different ages. Schematic representation of the psychiatric manifestations most commonly reported over the years in NCL patients and as disease progresses, illustrating how distinct symptom profiles emerge from early childhood to adulthood

Figure 3 schematically summarizes the predominant psychiatric phenotypes across NCL clinical subtypes and developmental stages. These manifestations can be conceptualized as evolving dimensional domains whose clinical expression varies according to age and disease stage. Emotional and behavioural dysregulation represents a frequent early manifestation across several NCL forms. In early childhood, these features are often nonspecific and may resemble neurodevelopmental conditions. With advancing age and disease progression, symptom profiles tend to become more differentiated, encompassing mood disturbances, anxiety-related symptoms, obsessive–compulsive features, and, in some cases, psychotic phenomena. This evolution should not be interpreted as the emergence of primary psychiatric disorders, but rather as part of the broader neurodegenerative process affecting behaviour, affect regulation, and cognition.

With regard to CLN3 disease, Adams and colleagues proposed an inverse U-shaped trajectory of behavioural symptoms, suggesting initial worsening during early disease progression followed by a later decline, potentially related to severe motor impairment and global functional deterioration [10]. However, subsequent studies have not consistently confirmed this pattern, instead reporting heterogeneous trajectories, with some behavioural manifestations intensifying over time while others attenuate [12] and this highlighted considerable individual variability. Several potential modifiers of psychiatric manifestations have been proposed. Sex differences have been suggested in some cohorts [69], with females reportedly exhibiting more severe anxiety and attentional difficulties, as well as faster progression despite a later disease onset [7]. However, these findings are not consistent [9]. Divergent psychiatric profiles observed among siblings carrying the same mutation further suggest the contribution of additional modifying factors, potentially including epigenetic or post-translational mechanisms [65], although robust genotype–phenotype correlations have not been established to date [10] .

Isolated case reports have also provided insights into the longitudinal evolution of psychiatric symptoms. For example, a recent report described a 46-year-old woman with CLN13 whose clinical course illustrates the progressive psychiatric trajectory that may occur in adult-onset forms [14]. She initially developed depressive symptoms at age 37, which evolved within one year into a manic episode, followed by cognitive decline, parkinsonism, and ultimately catatonia, confirmed using the Bush–Francis Catatonia Rating Scale.

### Multifactorial drivers of psychiatric symptoms in NCLs

Psychiatric manifestations observed in NCLs likely result from a multifactorial interaction of neurobiological mechanisms, disease-related processes, and environmental influences. Their relative contribution varies across infantile- and juvenile-onset forms, depending on neurodevelopmental stage, rate of degeneration, and preserved cognitive insight. Progressive neuronal loss involving cortical and subcortical circuits disrupts networks regulating emotion, social behavior, and impulse control. In juvenile CLN3, degeneration within the anxiety/fear network - including the insula, anterior cingulate, amygdala, hypothalamus, and brainstem - has been linked to sympathetic hyperactivation, producing exaggerated fear and anxiety responses [35]. Clinically, this manifests as recurrent non-epileptic episodes of agitation, fearful behavior, tachycardia, diaphoresis, and hyperthermia, resembling paroxysmal sympathetic hyperactivity (PSH), a syndrome also seen in acquired brain injury [34]. Even in late disease stages, primitive fear reactions persist despite loss of anticipatory anxiety, suggesting a brainstem–hypothalamic origin. Additional factors further influence psychiatric symptoms, such as chronic discomfort (pain, spasticity, gastrointestinal issues) [34, 47] and sleep–wake disturbances contribute to irritability, anxiety, and mood problems [35, 47]. Cognitive decline is known to play a relevant role in behavioral manifestations. In juvenile forms such as CLN3, awareness of progressive decline can trigger reactive depression and anxiety, while in infantile forms the profound early neurodevelopmental impact makes such insight-driven responses less likely [34]. Family environment, family support, and pre-existing familial psychopathology may strongly modulate psychiatric symptoms in NCLs [13]. Progressive visual loss also precipitates psychiatric symptoms: in CLN3, visual “release” hallucinations, akin to Charles Bonnet syndrome, arise from deafferentation of the visual cortex and must be distinguished from primary psychosis [67]. Finally, therapies themselves may influence psychiatric status. For instance, a recent post-marketing pharmacovigilance study based on EudraVigilance data identified multiple suspected adverse reactions (SARs) to cerliponase alfa in patients with CLN2 disease [70]. While the majority of SARs were infusion-related or neurological (e.g., seizures, headache), several are referred to behavioral and emotional functioning [70]. Psychiatric disorders reactive to therapy accounted for 4.4% of total adverse reactions (14/321 SARs); irritability has been reported up to 6.1% of cases, at it seems more frequent in females [70]. These findings underscore the importance of systematic behavioral monitoring during cerliponase alfa therapy, both to detect potential adverse psychotropic effects and to differentiate them from disease-related psychiatric symptoms.

### Managing psychiatric symptoms in NCLs: current practice and pitfalls

The management of psychiatric and behavioral symptoms in NCLs requires a multidisciplinary approach. While no controlled trials are available, a combination of pharmacological and psychosocial strategies appears promising. Beyond medications, person-centered interventions may help reduce behavioral instability and caregiver burden. For example, in a recent CLN3 case study, a tailored psychosocial program led to marked improvements in irritability, emotional lability, and caregiver–patient interactions, with reductions in NPI-Q scores for severity and distress [13]. Although anecdotal, such findings highlight the importance of structured routines, sensory engagement, and individualized psychological support.

Pharmacological management remains largely empirical. Available evidence on the use of psychotropic medications in NCLs is largely anecdotal and primarily derived from case reports and small surveys conducted in CLN3 disease. Data for other NCL subtypes is even more limited or entirely lacking. Case reports in adult-onset forms (e.g., Kufs disease) describe the use of antipsychotics for psychosis-like symptoms, though efficacy is inconsistently reported and side effects often limit tolerability. A survey of Finnish JNCL patients [58] identified Citalopram as the most frequently used antidepressant for affective symptoms, and Risperidone, Olanzapine, or Quetiapine for psychotic manifestations, with variable but generally satisfactory outcomes. Citalopram has also been used successfully to treat OCD symptoms in patients with CLN6-related disease [71]. Atypical antipsychotics and SSRIs therefore remain the mainstay of practice [58, 72, 73]. However, patients with NCL appear especially vulnerable to motor side effects, likely reflecting degeneration within dopaminergic system [74, 75]. In this context, agents with partial dopamine D2 agonism and serotonergic modulation (e.g., Aripiprazole) may offer a safer profile, although side effects are common too. Preclinical evidence further suggests potential disease-modifying properties of Aripiprazole through lysosomal storage reduction in both in vitro and in vivo models of CLN3 and CLN7 diseases [72], though there is impact on sleep, concentration and gastrointestinal manifestations. Tobo and colleagues [44] described a patient initially diagnosed with schizophrenia who had received a total of 95 ECT treatments over the course of his lifetime with apparently “slight improvements” noted at times. Serious adverse reactions, including neuroleptic malignant syndrome (NMS) [36, 61, 73] and serotonin syndrome with SSRIs [73], have been reported even at low doses [61]. Valproic acid is frequently prescribed for both myoclonus, epilepsy and mood instability, but hyperammonemia [76] and acceleration of motor decline in CLN2 disease [77] require careful monitoring. For mood stabilization, Lithium salts may represent a rational alternative, supported by experimental data showing restoration of defective autophagy in CLN3 models [78]. For anxiety related to autonomic dysregulation, non-standard agents such as gabapentin, benzodiazepines, clonidine, or propranolol have been proposed, though systematic evaluation is lacking [79]. Although the available evidence is insufficient to define specific drug selection or dosage regimens, Supplementary Tables S3 and S4 provide a quite exhaustive overview of psychotropic medications reported in the literature. These tables are not intended as treatment recommendations but rather as a synthesis of the limited and low-quality data — largely derived from case reports and small series — currently available on the management of psychiatric manifestations in NCLs.

**Fig. 4:**
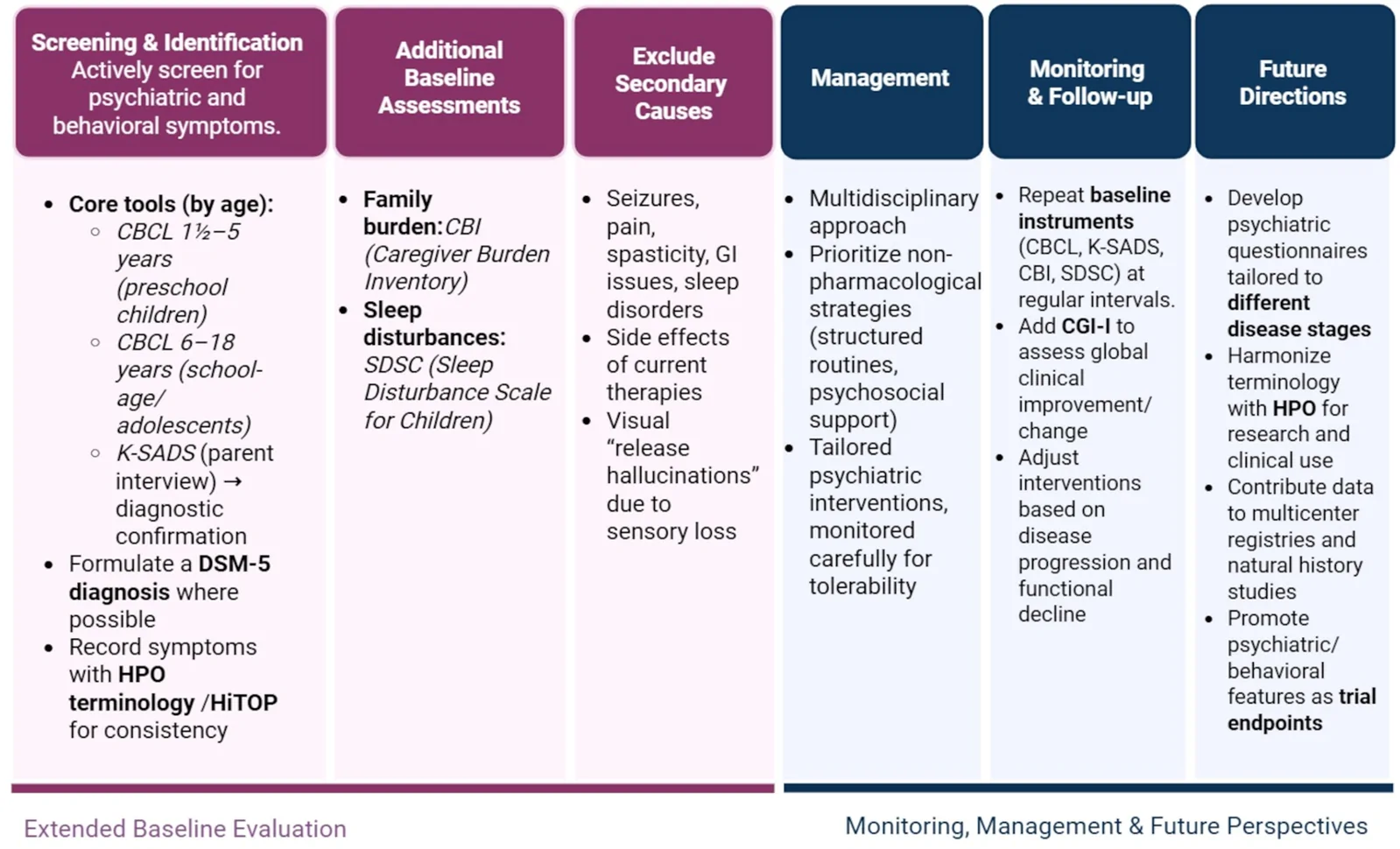
Practical flowchart for the assessment and management of psychiatric manifestations in NCLs. To address heterogeneous reporting and the lack of standardized guidance, the flowchart outlines a stepwise approach that (1) screens and identifies symptoms using age-appropriate tools; (2) adds baseline assessments of caregiver burden (CBI) and sleep disturbances (SDSC); (3) excludes secondary causes; (4) guides management via a multidisciplinary approach that prioritizes non-pharmacological strategies and carefully monitored, individualized psychiatric interventions; (5) recommends monitoring and follow-up with regular re-administration of baseline instruments, addition of CGI-I to gauge global improvement/change, and adjustment of interventions to disease progression and functional decline; and (6) highlights future directions, including harmonization of terminology with HPO for clinical and research use, contribution to multicentre registries and natural-history studies, and promotion of psychiatric/behavioral features as trial endpoints

In the absence of expert guidelines and with heterogeneous clinical manifestations, we propose a practical flowchart describing our personal approach to assessment and management of psychiatric manifestations in NCLs. With the limitations of a single user experience, these practical recommendations could assist while integrating psychiatric outcomes in clinical practice and research (Fig. 4). The proposed assessment strategy incorporates age-appropriate and clinically feasible instruments selected according to symptom profile. Regarding psychiatric assessment, we selected the ASEBA Child Behavior Checklist (CBCL) [80], according to the patient’s age, as a broad screening tool, as it has already been used in NCL cohorts and has shown correlation with disease-specific measures, including the UBDRS [10]. Furthermore, we proposed clinical confirmation using the Kiddie Schedule for Affective Disorders and Schizophrenia (K-SADS) [81], where feasible, to provide a more quantitative psychiatric assessment and, when appropriate, support DSM-based diagnostic classification. In addition, we emphasize the potential value of standardized terminology through Human Phenotype Ontology (HPO) descriptors to improve harmonization of psychiatric and behavioral phenotyping across clinical care, natural history studies, and future multicentre trials. We also considered the assessment of sleep, as sleep disturbances may worsen psychiatric symptoms and increase caregiver burden. For this purpose, we proposed the Sleep Disturbance Scale for Children (SDSC), a widely used and validated parent-reported questionnaire in pediatric populations [82]. Finally, we considered it important to assess caregiver burden, given the significant impact that psychiatric symptoms may have on family well-being. For this reason, we included the Caregiver Burden Inventory (CBI) [83] as part of the proposed framework. This latter instrument was originally developed for adult populations, particularly in dementia care, but has also been used in pediatric settings [84].

Importantly, this framework is particularly applicable to patients in the early stages of the disease, when psychiatric symptoms are more accessible to standardized assessment and potentially more responsive to intervention. In advanced stages, however, adaptations are required, including simplified evaluation tools, greater reliance on caregiver-based observations, and symptom-focused rather than syndrome-based management strategies.

### Psychiatric symptoms as potential endpoints in NCL trials

In the current therapeutic era, characterized by the rapid expansion of disease-modifying approaches, the identification of robust, sensitive, and clinically meaningful outcome measures has become an urgent priority. Within this framework, psychiatric manifestations should no longer be viewed as ancillary features of NCLs, but rather as measurable and potentially modifiable dimensions of disease burden. Integrating these domains into clinical trials requires a systematic effort to define reliable assessment strategies capable of detecting subtle yet meaningful changes over time. Current clinical trials primarily rely on motor, cognitive, or survival metrics, which capture only part of the disease burden and may underestimate treatment benefits that are meaningful to patients and families. Psychiatric outcomes integration into clinical research remains limited by the absence of standardized, calibrated instruments capable of capturing subtle behavioral changes over time, also in the late stage of the disease. Recent consensus recommendations from the *Batten Disease Clinical Research Consortium* [85] have identified the definition of sensitive, patient-centered outcome measures as a top research priority for the NCL field. In this context, the systematic validation of psychiatric and behavioral tools — through longitudinal, multicenter studies — is crucial to establish normative trajectories, determine measurement reliability, and identify the endpoints most responsive to intervention. Developing such measures would allow more precise evaluation of treatment efficacy, complement established neurological parameters, and expand the concept of therapeutic benefit beyond survival and motor function to encompass mental health, adaptation, and overall quality of life for affected children and their families.

## Discussion

This review aimed to summarize the state of the art of psychiatric symptoms in NCL disorders. The first important point is that psychiatric manifestations are common across all NCL subtypes and ages. The spectrum of psychiatric problems is broad. In infantile and late-infantile forms, emotional and behavioral problems are the most common reported with traits of neurodevelopmental disorders [5, 15–18]. In Juvenile forms, attentional problems and social withdrawal progresses to anxiety, depression, and obsessive–compulsive symptoms in adolescence, with psychotic features emerging later [7, 9, 32, 35, 39]. In adult-onset forms, psychosis may even be the first sign [5, 7, 9, 19, 36–44]. This age-related sequence supports a developmental–neurodegeneration continuum model, in which the timing and distribution of neuronal loss interact with ongoing brain maturation. Such trajectories also suggest that the psychiatric phenotype of NCLs is not static but dynamic across development, shaped by neural plasticity, residual function, and compensatory mechanisms. Additional modifiers - including genotype, epigenetic regulation, sex, and family environment - likely contribute to this heterogeneity. Understanding these modifiers is crucial not only for pathophysiological insight but also for refining trial stratification and predicting treatment response.

Another important point is the multifactorial nature of these manifestations, depending on the extent and distribution of neurodegeneration, autonomic and sensory dysfunction, cognitive decline, genetic and epigenetic factors, psychosocial environment and so on. Such factors dynamically interact over time, amplifying or modulating symptom expression and contributing to the marked clinical heterogeneity observed across and within subtypes.

The management of psychiatric symptoms in NCLs remains largely empirical. Psychotropic medications are used for palliative care, yet side effects and the potential for paradoxical worsening underscore the need for cautious, individualized use. Some psychiatric agents (e.g., Lithium [78], Fluoxetine [86]) seem to modulate autophagy pathways and therefore deserve further investigation as potentially disease-relevant treatments to be consider beyond their use for symptomatic control. Non-pharmacological strategies, such as structured routines, behavioral therapy, and tailored psychosocial support, offer meaningful benefits in daily functioning and caregiver well-being but require formal evaluation through standardized outcomes [13].

Given the high prevalence of psychiatric disturbances in pediatric NCLs, standardized psychiatric assessment should become a mandatory component of both routine care and longitudinal monitoring. The diagnostic overlap between seizure-related phenomena (such as fear orautonomic episodes) and psychiatric presentations (such as panic attacks) further reinforces the need for structured clinical algorithms, such as the proposed flowchart, to guide differential diagnosis and follow-up.

Current research priorities for NCLs include identifying optimal treatment strategies for dementia and behavioral challenges and developing robust trial designs. This includes defining ideal study populations, appropriate durations, clinical endpoints, and biomarkers [85]. The disease burden is only partially captured by traditional endpoints (e.g., motor scales, survival). Psychiatric symptoms, on the other hand, reflect patient-centered dimensions such as emotional regulation, social adaptation, and quality of life. Their temporal sensitivity and developmental predictability make them particularly suitable as dynamic outcome measures.

Psychiatric assessments have already been incorporated into natural history studies and clinical trials in several pediatric-onset neurodevelopmental and neurogenetic disorders. For example, the CBCL has been used in the Natural History Study of Mucopolysaccharidosis III (ClinicalTrials.gov ID: NCT02037880) and in studies involving adolescents with Fragile X syndrome and autism spectrum disorder (ClinicalTrials.gov ID: NCT06677866). Other instruments are commonly selected according to the target symptom domain, like the Aberrant Behavior Checklist (ABC) is frequently used to assess irritability, hyperactivity, lethargy/social withdrawal, stereotypic behavior, and inappropriate speech [87]; Repetitive Behavior Scale-Revised (RBS-R) [88] are used to characterize syndrome-specific behavioral, social-communication, and repetitive-behavior domains; the social communication questionnaire (SCQ) [89] and Autism Diagnostic Observation Schedule (ADOS) [90] for autism. Clearly, a comprehensive discussion of all psychiatric instruments potentially applicable to paediatric populations is beyond the scope of the present review. In the present paper, we particularly focused on the CBCL because it has already been applied in NCL populations, and its correlation with the UBDRS has previously been demonstrated [10], supporting its potential utility as a broad screening instrument in this setting. Given the severity and progressive nature of NCLs, broad-spectrum tools such as the CBCL may therefore represent a pragmatic first step to define the psychiatric spectrum before refining more specific outcome measures for future clinical trials.

On the other hand, it is important to highlight that many currently available psychiatric assessment tools in pediatric neurodevelopmental disorders are largely caregiver- or parent-reported. While this approach is often necessary in severely impaired populations such as NCLs, it may reduce the ability to accurately capture internally experienced symptoms, including anxiety, depressed mood, obsessive thoughts, hallucinations, or other subjective psychiatric experiences. This limitation should be carefully considered in the development of future psychiatric outcome measures for clinical trials.

Using precise and standardized terminology is essential for the characterization of psychopathological phenotypes, whether one relies on DSM-5 categorical diagnoses, dimensional frameworks such as Hierarchical Taxonomy of Psychopathology (HiTOP), or phenotypic ontologies like Human Phenotype Ontology (HPO). It is critical to ensure comparability across cohorts and research studies. This can be accomplished through harmonization through standardized instruments (e.g., CBCL) and shared terminologies — DSM-5, HPO, and HiTOP. Importantly, reducing the clinical presentation of highly complex patients to a single diagnostic label can be extremely challenging, if not misleading. In such cases, a dimensional approach may be more appropriate, as it captures the breadth and variability of symptoms more accurately than a strict categorical framework. This precision in language and conceptualization is not a mere technicality but a prerequisite for valid cross-study integration, replicability, and cumulative scientific progress. For this reason, future effort should validate age-appropriate, sensitive instruments for psychiatric assessment, establish longitudinal trajectories linking psychiatric manifestations to neurobiological and imaging (or even fluid) biomarkers, and test whether targeted interventions, pharmacological or psychosocial, can modify both symptoms and disease course. By integrating psychiatric endpoints into trial design, the field can move beyond descriptive psychopathology toward quantifiable, clinically meaningful measures of patient well-being, expanding the therapeutic focus of NCL research to include mental health and adaptive functioning as core dimensions of disease burden, alongside survival and neurological outcomes.

## Supplementary Information

Below is the link to the electronic supplementary material.


Supplementary Material 1


## Data Availability

Data source and materials are available from the corresponding author (F.M.S) on the basis of reasonable request.

## Abbreviations

ANCL: Adult Neuronal Ceroid Lipofuscinosis
ASD: Autism Spectrum Disorder
CBCL: Child Behavior Checklist
CBI: Caregiver Burden Inventory
CDI: Children’s Depression Inventory
CGI-I: Clinical Global Impression–Improvement
CLN: Ceroid Lipofuscinosis, Neuronal (gene-based subtype classification: CLN1–CLN14)
CY-BOCS: Children’s Yale–Brown Obsessive Compulsive Scale
DSM-5: Diagnostic and Statistical Manual of Mental Disorders, Fifth Edition
ECT: Electroconvulsive Therapy
HPO: Human Phenotype Ontology
HiTOP: Hierarchical Taxonomy of Psychopathology
JNCL: Juvenile Neuronal Ceroid Lipofuscinosis
INCL: Infantile Onset Neuronal Ceroid Lipofuscinosis
LINCL: Late Infantile Onset Neuronal Ceroid Lipofuscinosis
vLINCL: Variant Late-Infantile Neuronal Ceroid Lipofuscinosis
NCL: Neuronal Ceroid Lipofuscinosis
NMS: Neuroleptic Malignant Syndrome
NPI-Q: Neuropsychiatric Inventory Questionnaire
PRISMA: Preferred Reporting Items for Systematic Reviews and Meta-Analyses
PSH: Paroxysmal Sympathetic Hyperactivity
SARs: Suspected Adverse Reactions
SDSC: Sleep Disturbance Scale for Children
SIB-R: Scales of Independent Behavior–Revised
SSRIs: Selective Serotonin Reuptake Inhibitors
TRF: Teacher’s Report Form
UBDRS: Unified Batten Disease Rating Scale

## Glossary

Neuronal Ceroid Lipofuscinoses (NCLs): Rare genetic lysosomal storage disorders causing progressive neurodegeneration, vision loss, seizures, and psychiatric symptoms; also called Batten disease
DSM-5: Standard classification system for psychiatric disorders
HPO (Human Phenotype Ontology): Structured vocabulary for clinical and genetic phenotypes
HiTOP: Hierarchical Taxonomy of Psychopathology
Paroxysmal Sympathetic Hyperactivity (PSH): Episodes of excessive sympathetic activation (e.g., tachycardia, agitation)
Release hallucinations: Visual hallucinations occurring in patients with severe vision loss (e.g., Charles Bonnet–like)
Psychotropic medications: Drugs affecting mood, cognition, or behavior (e.g., antidepressants, antipsychotics)
Cerliponase alfa: Enzyme replacement therapy for CLN2 disease
Psychopathology: Study of mental and behavioral symptoms and their mechanisms
