# Supplementary material for: Psychiatric manifestations in Neuronal ceroid lipofuscinoses
Source: Orphanet J Rare Dis. 2026 Jun 15;21:303. doi: 10.1186/s13023-026-04416-0 (PMC13528172; doi:10.1186/s13023-026-04416-0)
Supplement: Supplementary file 1 [file 13023_2026_4416_MOESM1_ESM.doc]

**Supplementary Table S1.** Clinical studies investigating psychiatric manifestations in NCL.

| **Reference** | **Type of study** | **Population** | **Aim** | **Tools** | **Psychiatric symptoms: outcome** |
| --- | --- | --- | --- | --- | --- |
| Bäckman et al., 2005 | Cross-sectional observational study | CLN3 n = 27  Medicated group: 3 M, 10 F (mean age 15 y). Drugs most used: risperidone (5/13) and citalopram (8/13).  Non-medicated group: 10 M, 4 F (mean age 15.3 y) | To characterize psychiatric symptoms in patients with JNCL and compare patients by psychotropic treatment status and gender. | CBCL  TRF  CDI | High prevalence of psychiatric symptoms, especially social, thought, and attention difficulties, somatic complaints, aggressive behavior, and sleep/speech disturbances. 74% of patients scored in clinical or borderline range. Females and those on psychotropic medication had more symptoms. Low self-reported depressive symptoms on CDI. |
| Adams et al., 2006 | Cross-sectional baseline study (part of a longitudinal cohort) | CLN3 n = 26  13 M, 13 F  Mean age 12 y 3 m | To assess behavior and adaptive living skills in JNCL with standardized tools | CBCL  SIB-R  YBOCS | High prevalence of psychiatric symptoms: 19/24 scored in borderline or clinical range on CBCL Total Problems. Most frequent were thought disorder, social problems, attention deficits, and aggressiveness. OCD-like behaviors (e.g., obsessions, rituals, compulsions) were frequent but often not distressing to the child. Behavioral symptoms inversely correlated with adaptive functioning (SIB-R). |
| Adams et al., 2010 | Cross-sectional observational study | CLN3 n = 60  28 M, 32 F  Mean age 15 y 1 m | To examine genotype-phenotype correlation in patients with homozygous common deletion or compound heterozygous variants in *CLN3*.  To cross-validate the CBCL and the UBDRS | CBCL  UBDRS | No significant differences in psychiatric symptom severity between genotypes. Strong correlation between UBDRS behavioral ratings and CBCL scores supports validity of both tools. Psychiatric and physical symptoms appeared independent. Most common behavioral symptoms included mood disturbances (apathy, sadness, anxiety), aggressiveness (toward self and others), and compulsive behaviors. |
| Backman et al., 2001 | Longitudinal prospective study – open-label pilot study | JNCL n = 14  receiving psychotropic drug treatment with citalopram, risperidone, olanzapine or quetiapine. | To evaluate parent-reported efficacy of psychotropic drugs in Finnish patients with JNCL. Follow up 3–5 months | Clinical interviews, physician and parent consensus assessments, medical records | 70% had good or satisfactory clinical response to the new psychotropic drugs. Most common symptoms treated: psychosis, affective symptoms, anxiety. Citalopram and risperidone were most used. Adverse effects included fatigue, weight gain, and exacerbation of extrapyramidal symptoms. No major adverse events reported. |
| Honingh et al., 2022 | Longitudinal retrospective study | CLN3 n = 11 | To explore the emotional and behavioral symptoms of children with CLN3 and examine the support received by children and families | Clinical evaluation, parent interviews, UBDRS | Anxiety was present in all children; other symptoms included sadness, aggressiveness, compulsive and stereotypical behaviors, hallucinations, and obsessive traits. Symptoms varied per individual. Emphasis on impact on family and unmet support needs. |
| Cialone et al., 2012 | Longitudinal prospective study | CLN3 n = 83  39 F, 44 M | To determine gender-dependent differences in JNCL | UBDRS, PedsQL, parent reports, Batten Disease Support and Research Association database | Behavioral symptoms (e.g., irritability, aggressiveness, sleep problems) began significantly earlier in males. However, females showed more rapid functional decline and lower physical quality of life. Psychiatric symptoms contributed to earlier impairment of ADL in females. Sex differences may relate to immune or hormonal factors, or gendered caregiving patterns. |
| Nickel et al., 2018 | Longitudinal prospective study | 140 patients with genetically confirmed CLN2 from two international datasets (DEM-CHILD and WCMC); longitudinal motor-language data available in 67 | To describe the natural history and clinical progression of untreated CLN2 using standardized motor-language scales | Clinical evaluation | Psychiatric symptoms were not the primary focus but behavioral abnormalities were among the early symptoms (16% of patients). Data highlight the predictability of psychomotor decline and underscore the need for early recognition of behavioral or language signs to prompt earlier diagnosis. |
| Lourenço et al., 2021 | Cross-sectional retrospective study | Atypical form of CLN2  n = 30 | To analyze clinical data from a cohort of Latin American CLN2 patients with atypical phenotypes | Clinical evaluation | Behavioral abnormalities were among the first symptoms in 7% of cases, and reported during the disease course in 56%. The most frequent were irritability, aggressiveness, and other unspecified behavioral disorders. These showed later onset than motor symptoms and seizures, but often co-occurred with cognitive decline and language regression. EEG was abnormal in 60%, with low-frequency photostimulation sensitivity in 37%. |
| Kravljanac et al., 2022 | Longitudinal retrospective study | CLN2 n = 22 | Phenotype characterization | Clinical evaluation | Behavioral problems were present in 14/22 patients in early stages and considered among the early “red flags” alongside language delay. These behavioral symptoms were part of the prodromal phase and associated with diagnostic confusion. While not the primary focus, behavior was a key early feature preceding seizure recurrence and progression. |
| Pérez-Poyato et al., 2011 | Longitudinal retrospective study | vJNCL CLN1 n = 11  cJNCL CLN3 n = 13 | Phenotype characterization | Clinical evaluation | Behavioral disturbances (e.g., irritability, autistic behavior, bradypsychia) occurred significantly more frequently, and earlier, in vJNCL patients. Median age at onset of behavioral disturbances: vJNCL, 8 y; cJNCL, 10 y. Autism-like features and mental retardation also appeared earlier in vJNCL. The findings suggest that psychiatric symptoms are early and discriminative features in subtype differentiation. |
| Simonati et al., 2009 | Longitudinal retrospective study | CLN1 n = 6  (late-infantile form) | Phenotype characterization | Clinical evaluation | Behavioral disturbances were among the earliest signs in 3 of 6 patients, including aggressiveness, stereotyped movements, oppositionality, and hallucinations. These symptoms appeared before or alongside motor decline and seizures. All patients had progressive cognitive and behavioral regression, with EEG showing early background slowing and loss of sleep spindles. Psychiatric symptoms were prominent in early disease and worsened over time. |
| Simonati et al., 2017 | Longitudinal retrospective study | CLN5 n = 15  9 M, 6 F  Age range 9–26 y | Phenotype characterization and natural history study | Quantitative scale assessing six domains (motor, vision, language, behavior, learning and cognition, and epilepsy) | Behavioral disturbances (hyperactivity, aggressiveness, motor stereotypes, anxiety, hallucinations, obsessive behavior) were observed in 11/15 patients, with onset at diagnosis in four cases. Behavioral symptoms often preceded seizures and visual loss, and were associated with faster decline in those with severe genotypes. Sleep disturbances were also reported in 9/15 patients. |
| Santavuori et al., 1993 | Cross-sectional, retrospective and survey-based study | JNCL two series: A) n=42, B) n= 13 | To describe the frequency, onset, and management of psychological and sleep disturbances in different NCL subtypes | Caregiver surveys, medical records, structured symptom checklists | Psychological symptoms were common in JNCL: 45% fear, 40% aggressiveness, 35% restlessness, 10% hallucinations. Symptoms started at a mean age of 10.2 y and persisted for years. Sleep disturbances (e.g., insomnia, nightmares, awakenings) were present in 30/55 patients. Symptoms in INCL often began earlier (1–3 years). Management included medication (e.g., baclofen, levomepromazine), psychosocial strategies, and environmental adjustments. |
| Marshall et al., 2005 | Cross-sectional study | CLN3 n = 31 | To develop and validate a standardized clinical scale (UBDRS) for assessing motor, behavioral, and functional impairment in JNCL | UBDRS | Behavioral domain included symptoms such as apathy, anxiety, aggression, stereotypies, obsessions, and hallucinations, rated by frequency and severity. Interrater reliability for behavioral scores was good (intraclass correlation coefficient = 0.68). Behavioral symptoms were common, often emerged after visual loss and seizures, and were considered central in disease monitoring. |
| Schulz et al., 2024 | Observational, cross-national survey and interview study | CLN3 parents (27 mothers, 7 fathers, 5 cases with unreported parental gender) | To investigate CLN3 disease progression, clinical care, and family experiences | Semi-structured interviews (85-question survey), qualitative and quantitative analysis | High prevalence of behavioral symptoms (74.4%) with onset around age 6.3 y; OCD (65.1%), anxiety (69.8%), and depression (39.5%) were frequently reported by caregivers. Insomnia (86%) was the most prevalent symptom after visual loss. Psychiatric symptoms were perceived as impactful and often under-addressed, with emotional and mood disturbances reported as a top concern. |
| Saleh et al., 2024 | Cross-sectional retrospective study | CLN1 (n = 4), CLN2 (n = 2), CLN3 (n = 1), CLN5 (n = 5), CLN6 (n = 25), CLN7 (n = 8), CLN8 (n = 8),  CLN14 (n = 2) | Cross-sectional study | Phenotyping description | Behavioral disturbances (hyperactivity, aggressiveness, sleep disturbance, depressed mood) were observed in 20% of patients, most frequently in CLN5 (57%). Average age at onset 3.6 y. Symptoms were often present early and worsened over time. Sleep disorders and psychiatric symptoms were not systematically assessed but noted in clinical data. |
| Paiva et al., 2023 | Longitudinal retrospective study | CLN5 n = 17  5M, 12F  Age range 1–40 y | Phenotype and molecular characterization | Clinical and molecular evaluation | Behavioral or psychiatric symptoms were not the central focus, but phenotypic variability was emphasized. One patient had adult-onset disease, expanding the expected age window. Neurodevelopmental regression and cognitive decline were universally present. No details on specific psychiatric manifestations (e.g., irritability, mood, compulsions) were provided. The study underlines interindividual variability and atypical onset features. |
| Valadares et al., 2011 | Longitudinal retrospective study | CLN3 n = 10 | To describe the clinical and molecular features and intrafamilial phenotypic variability of JNCL in a large Brazilian kindred | Clinical evaluation | Behavioral alterations were present in several patients, including autistic behaviors, tics, temper tantrums, and aggressive outbursts. One patient had a particularly severe phenotype with autism, tics, and parkinsonism. Behavioral symptoms emerged early in some patients and preceded seizures or motor signs. Wide phenotypic variability was observed even within the same family. |
| Sakti et al., 2023 | Cross-sectional study | CLN3 n = 5  4F, 1M  median age 6.2 y | Phenotype characterization | Clinical evaluation | Neurobehavioral symptoms were present in 4 of 5 patients, including autistic traits, anxiety, and behavioral abnormalities. Although not the primary focus, these symptoms were considered part of the disease spectrum and noted in clinical observations. |
| Larsen et al., 2014 | Cross-sectional study | CLN3  35 patients with CLN3, of whom  14 treated with VPA | To describe four cases with valproate-induced hyperammonemia in a well-defined Danish JNCL population | Clinical assessment | Psychiatric symptoms including confusion, irritability, personality change, and lethargy were reported in patients with valproate-induced hyperammonemia. The authors highlight need for caution with valproate use in JNCL due to psychiatric vulnerability and polytherapy. |
| Kamate et al., 2012 | Longitudinal retrospective study | CLN1 n = 5  CLN2 n = 11 | Phenotype characterization | Clinical evaluation | Behavioral symptoms such as irritability and autistic features were observed especially in CLN1 patients (e.g., in a child with speech delay and autistic traits). These symptoms often appeared early, but were under-recognized due to overlapping neurological signs. Behavioral data were not systematically collected. The authors suggest increased attention to early neuropsychiatric signs to reduce diagnostic delay. |
| Topçu et al., 2004 | Longitudinal retrospective study | INCL n =3  LINCL n = 11  JNCL n = 5  Turkish variant NCL n = 17 | Phenotype characterization | Clinical evaluation | Progressive mental regression, personality changes, speech impairment, autistic behavior, severe seizures; Turkish variant showed more severe neuropsychiatric progression and earlier visual and behavioral abnormalities than other types. |
| Mandel et al., 2000 | Longitudinal retrospective study | CLN7 n = 5  2F, 3M | Phenotype characterization | Clinical evaluation | Progressive cognitive and behavioral decline: early-onset seizures (age 5–7), regression of social and cognitive skills (age 8–10), aggressive and unsociable behavior, leading to vegetative state by second decade. |
| Ranta et al., 2000 | Longitudinal retrospective study | CLN8  n = 26 | Phenotype characterization | Clinical evaluation | Progressive mental retardation 2–5 y after epilepsy onset (age 5–10); behavioral problems in childhood and puberty (irritability, restlessness, disobedience, inattentiveness) |
| Velinov et al., 2012 | Longitudinal retrospective study | ANCL  (autosomal dominant Kufs disease)  n = 9 | Phenotype characterization | Clinical evaluation | Psychiatric symptoms were often the first manifestations (e.g., obsessive–compulsive traits, irritability, anxiety); followed by seizures, cognitive decline, and dementia. Onset usually in 20s–30s. Behavioral and psychiatric symptoms were prominent and appeared early in *DNAJC5*-positive cases. |
| Savvidou et al., 2022 | Longitudinal retrospective study | CLN3 n= 4  3F, 1M  Median age 14.75 y | To describe the occurrence of drug-induced hyperthermia linked to NMS and SS caused by neuroleptic and serotonergic drugs in pediatric patients with CLN3 | Clinical evaluation | Severe psychiatric symptoms (e.g., hallucinations, anxiety, aggressiveness) led to treatment with psychotropic drugs, resulting in drug-induced hyperthermia, status dystonicus, and rhabdomyolysis. Three of the four patients died. Psychiatric symptoms were prominent and difficult to manage. |
| Khan et al., 2013 | Case report | atypical juvenile CLN1 | Phenotype characterization | Clinical evaluation | Visual hallucinations followed later by behavioral and cognitive decline. Psychiatric symptoms emerged late in the disease course. |
| Augustine et al., 1993 | Case report | ANCL  24-year-old female | Phenotype characterization | Clinical evaluation | Early symptoms: phobia, depression, disordered thinking, social withdrawal. Later symptoms: seizures, cognitive decline, choreiform movements, dysarthria. Psychiatric symptoms preceded seizures by several months. Diagnosis confirmed by brain biopsy. |
| Josephson et al., 2001 | Longitudinal retrospective study | Family with ANCL (n=10) | Phenotype characterization | Clinical evaluation | Psychiatric symptoms (dementia, hallucinations, disordered thinking) were an early and consistent feature. Dementia with seizures and motor abnormalities appeared by the 4th decade; progressive memory, executive, and visuospatial deficits were confirmed psychometrically. Dementia typically followed seizures within three years. |
| Hinkebein et al., 1997 | Case report | ANCL  49-year-old female | Phenotype characterization | Clinical evaluation | Early symptoms: paranoia, psychosis, suspicion, hallucinations, emotional flatness. Progressed to severe dementia, motor dysfunction, echolalia, disorganized speech. Psychiatric symptoms were among the first signs; initially misdiagnosed as primary psychiatric illness. |
| Cannelli et al., 2007 | Case report | CLN5 n =2  16-year-old boy  9-year-old boy | Phenotype characterization | Clinical evaluation | Early behavioral disturbances (age 5), cognitive decline before seizures (age 10–12), progressive mental deterioration, mild epilepsy. Psychiatric and cognitive symptoms were prominent and early. |
| Callagy et al., 2000 | Case report | ANCL  Two siblings: 38-year-old woman and 43-year-old man | Phenotype characterization | Clinical evaluation | Early dementia and behavioral changes as initial manifestations. |
| Iannetti et al., 1994 | Case report | LINCL | Phenotype characterization | Clinical evaluation | Progressive mental deterioration with visual and auditory hallucinations from age 11. |
| Craiu et al., 2015 | Case report | CLN7  7-year-old girl | Phenotype characterization | Clinical evaluation | Early loss of purposeful hand use, autistic features, behavioral regression, and lack of interaction, resembling Rett syndrome. Psychiatric features included autistic-like behavior and behavioral disturbances from age 2. |
| Kälviäinen et al., 2007 | Case report | juvenile-onset CLN1 | Phenotype characterization | Clinical evaluation | From age 6: hyperactivity, poor concentration (behavioral symptoms). From age 17: cognitive deterioration, delusions, attention deficits, speech reduction, loss of cooperation, severe psychomotor slowing. Progressive psychiatric decline into young adulthood. |
| Lee et al., 2010 | Case report | CLN3 n = 2  1 M, 1 F | Phenotype characterization by gender | Clinical evaluation | Female: auditory hallucinations, paranoid delusions, depression, emotional lability, cognitive impairment, seizures. Male: no psychosis or mood symptoms but showed behavioral dyscontrol, aggressiveness, tantrums, and social inappropriateness. Marked intrafamilial variability. |
| Lewandowska et al., 2009 | Case report | ANCL (CLN4)  45-year-old woman | Phenotype characterization | Clinical evaluation | Early symptoms: progressive dementia, psychosis, auditory and visual hallucinations, delusions, anxiety, aggressiveness, disinhibition, loss of social awareness, insomnia, speech disturbances. Profound psychiatric and behavioral alterations preceded neurological decline. |
| Reif et al., 2003 | Case report | ANCL  30-year-old woman | Phenotype characterization | Clinical evaluation | Initial presentation: behavioral disturbances and psychotic symptoms (catatonia, hallucinations, mutism, excitation). Later: delirium, paranoia, NMS after neuroleptic treatment. Persistent presenile dementia. Severe psychiatric symptoms preceded diagnosis and worsened with treatment. |
| Ozono et al., 2018 | Case report | Juvenile-onset CLN1 | Phenotype characterization | Clinical evaluation | Visual and auditory hallucinations at age 14; later psychosis, severe speech reduction, IQ 22, loss of interaction. Marked psychiatric and cognitive decline before seizures appeared. Final stage: mutism, severe atrophy, bedridden state. |
| Schaefers et al., 2021 | Case report | CLN2 n = 2 | Phenotype characterization | Clinical evaluation | Patient 1: early behavioral changes (frustration, crying, anger) and sleeping disorder, only partial response to medication. Patient 2: no behavioral or psychiatric manifestations reported. |
| Niida et al., 2016 | Case report | CLN1 n = 1 | Phenotype characterization | Clinical evaluation | From 12 months: irritability and sleeping problems. By 14–18 months: loss of social interaction, eye contact, gestures, and smiling; autistic-like regression. Later: severe psychomotor deterioration, hallucination-like hand stereotypies, sleep disturbance, vegetative state. |
| Madaan et al., 2020 | Case report | CLN2 | Phenotype characterization | Clinical evaluation | At 1.5 years: autistic regression with loss of social/communication skills, repetitive behaviors, restricted interests. Persistence of autistic features despite interventions. Later: epilepsy, ataxia, drooling. No improvement in autistic symptoms; psychiatric/behavioral phenotype dominated the early course. |
| Pasquetti et al., 2023 | Case report | CLN7  5-year-old girl | Phenotype characterization | Clinical evaluation | Behavioral/psychiatric: mild abnormalities, including poor interest in peers and occasional motor stereotypies. Moderate intellectual disability. Progressive developmental regression with psychomotor delay, bradykinesia, balance disturbance, epilepsy, and severe visual loss. |
| Kulkarni et al., 2021 | Case report | CLN13  39-year-old woman | Phenotype characterization | Clinical evaluation | Onset with psychosis: paranoid delusions, auditory hallucinations, withdrawal. Resistant schizophrenia (poor response to multiple antipsychotics). Seizures developed after clozapine. Later: progressive cognitive decline → frontotemporal dementia (memory loss, apraxia, reduced speech, dependency). |
| Vercammen et al., 2003 | Case report | CLN3  23-year-old woman | Phenotype characterization | Clinical evaluation | Longstanding psychiatric symptoms: inappropriate behavior (laughing, weeping, echolalia), agitation, and frequent paranoid hallucinations. Treated with risperidone (0.5 mg/day), developed NMS (rigidity, hyperthermia, autonomic instability). Behavioral/psychiatric symptoms were major management challenge. |
| Waldman et al., 1992 | Case report | ANCL  37-year-old woman | Phenotype characterization | Clinical evaluation | Psychiatric onset with confusion, anxiety, misdiagnosed as multiple personalities, later psychosis with cognitive decline. These findings highlight psychiatric/behavioral changes as first manifestations of neurodegenerative disease. |
| Tobo et al., 1984 | Case report | ANCL  49-year-old man and 51-year-old woman | Phenotype characterization | Clinical evaluation | Predominant psychiatric/behavioral symptoms: episodic psychotic states, stupor, hallucinations, delusions, irritability, violent/destructive behavior. Both cases misdiagnosed as schizophrenia, underwent repeated ECT. Later developed dementia, seizures, stuporous states, and became bedridden. Psychiatric manifestations were the initial and predominant features. |
| Zini et al., 2008 | Case report | ANCL  37-year-old woman | Phenotype characterization | Clinical evaluation | At onset (37 y): memory deficits and behavioral changes (apathy, disinhibition, untidiness). Later: visual hallucinations, agitation, wandering during seizures. Progressed to frontotemporal dementia with apraxia, anomia, poor comprehension. Psychiatric/behavioral changes were early and prominent. |
| Vital et al., 1991 | Case report | ANCL | Phenotype characterization | Clinical evaluation | Case 1 (male): onset with personality and social conduct changes → later: stereotyped behaviors, hyperorality, temporospatial disorientation, anosognosia, speech limitation with echolalia → total aphasia, dementia. Case 2 (female): onset with speech apraxia, anxiety, depression, memory impairment → later: aphasia, apraxia, amimia, oculomotor disturbances, dementia. Both had early psychiatric/behavioral disturbances as part of disease onset. |
| Johannsen et al., 2016 | Case report | CLN2 n = 2 | To describe two cases of VPA-induced movement disorder in later stages of late-infantile CLN2 | Clinical assessment | Both patients developed status dystonicus, hyperthermia, hyper-CK-emia, and altered consciousness while on VPA. After discontinuation of VPA, rapid improvement. No primary psychiatric or behavioral features described; neuropsychiatric outcome dominated by encephalopathy secondary to drug–disease interaction. |
| Kulsirichawaroj et al., 2022 | Case report (2 patients) | Two female pediatric patients diagnosed with CLN1 and CLN6 | To describe two cases presenting with Rett-like phenotype and to highlight the importance of differential diagnosis | Genetic testing (*MECP2*, *PPT1*, *CLN6*), electron microscopy of skin biopsy, clinical neurological examination | Both patients presented with Rett-like features, including hand-wringing and developmental regression. The study highlights that CLN1 and CLN6 can mimic Rett syndrome, emphasizing behavioral overlap and the importance of screening for NCL in *MECP2*-negative cases with regression and stereotypies. |
| Kozina et al., 2018 | Case report (1 patient) | 5-year-old Russian girl with NCL type 7 (*MFSD8* mutation) | To report clinical and genetic findings in the first Russian case of NCL7 caused by a novel *MFSD8* mutation | Clinical evaluation, *MECP2* gene testing, WES | The patient presented with Rett-like features including developmental regression, stereotypies, and loss of ambulation. Initially suspected to have Rett syndrome. Behavioral features such as stereotyped movements and cognitive deterioration were prominent and contributed to diagnostic confusion with classic neurodevelopmental disorders. |
| Ammendolia et al., 2024 | Longitudinal retrospective study | CLN2; 148 patients with suspected adverse reactions; 58.1% female, 41.9% male; age ranges: 2 m–2 y (5.8%), 3–11 y (70.9%), 12–17 y (19.4%), 18–64 y (3.9%) | To compile clear information and improve knowledge on the safety profile of cerliponase alfa using EudraVigilance spontaneous reporting data | Clinical evaluation | Psychiatric disorders accounted for 4.4% of total adverse reactions (14/321 SARs); irritability was reported (6.1% of SARs), more frequent in females; no conclusion was reached on long-term psychiatric progression. |
| Atee et al., 2024 | Case report | CLN3, female, 20 years old | To describe behaviors and psychological profile, contributing factors, and the effectiveness of person-centered psychosocial interventions | NPI-Q, PainChek® | NPI-Q total domain score reduced from 6 to 2; distress rating from 11 to 8; severity score from 10 to 6. Reduction in irritability/lability, improved behavior with routine, sensory engagement, and tailored caregiver approach. |
| Privitera et al., 2023 | Case report / family genetic study (longitudinal retrospective) | Large family: 12 members affected by bipolar disorder across five generations. Detailed study of eight individuals — 3 with BD-I or BD-II (1 M 63 y, 1 F 55 y, 1 F 27 y), 1 “borderline” (F 30 y), 4 unaffected. CLN type: heterozygous *CLN6* variant carriers; some also with *ZNF92* variant | To describe the clinical and genetic characteristics of a family with bipolar disorder and to identify genetic variants associated with disease predisposition | DSM-5 criteria, Mood Spectrum-Self Report (Lifetime Version), Clinical Global Impression, Montgomery–Åsberg Depression Rating Scale, Young Mania Rating Scale. | Genetic findings suggest *CLN6* variant predisposes to obsessive–compulsive traits; combined with *ZNF92* variant → more severe bipolar phenotype. |
| Ostergaard JR, 2018 | Longitudinal retrospective study | Five patients with CLN3; both genders; followed up for 15 years | To substantiate that paroxysmal non-epileptic attacks resembling paroxysmal sympathetic hyperactivity also occur in JNCL patients | Clinical observation; heart rate variability studies; EEG (to exclude epileptic activity) | Episodes of anxiety/agitation with frightened facial expression, excessive sweating, increased body temperature, blood pressure, heart and respiratory rates, and involuntary motor activity; no epileptic discharges; symptoms often triggered by non-nociceptive or mildly nociceptive stimuli; attacks ceased during sleep; progressive reduction of parasympathetic activity with age leading to autonomic imbalance. |
| Lanska et al., 1993 | Case report | Male, CLN3, onset at 5 y | To describe the clinical features of visual “release hallucinations” in a child with JNCL and differentiate them from psychosis, dementia, and seizure-related hallucinations | Clinical observation; ophthalmologic exam; neuropsychological test (WISC-R verbal IQ); electroretinogram; MRI; SPECT; EEG; CSF exam | Persistent formed and unformed visual hallucinations unrelated to seizures; hallucinations began with blindness, were novel and varied; no improvement with antiepileptic treatment; not explained by psychosis or dementia. |
| Bayir et al., 2025 | Case report | 46-year-old woman with *CTSF*-related CLN13 | To describe a case initially diagnosed as bipolar disorder who later developed dementia, parkinsonism, and catatonia, leading to NCL13 diagnosis | Clinical exam, psychiatric evaluation, Bush–Francis Catatonia Rating Scale, MRI, FDG-PET, CSF biomarkers, WES | Initial depressive and manic episodes → bipolar diagnosis; progressive behavioral changes, cognitive decline, social withdrawal; later parkinsonism and catatonia confirmed with lorazepam challenge and Bush–Francis scale; psychiatric course showed overlap with mood disorder and dementia features. |
| Özkara et al., 2017 | Retrospective case report | Two adult Turkish siblings with adult-onset CLN6 | To describe the temporal evolution of clinical manifestations, electrophysiological features, and treatment response in *CLN6*-related adult-onset Kufs type A disease | Clinical evaluation | Neither patient developed cognitive decline, but both showed significant psychiatric symptoms, particularly OCD and depression (clearly documented in Patient 1). Psychiatric symptoms were persistent throughout the disease course but did not show the same progression as neurological symptoms; Patient 1 received citalopram, with reported benefit. |

NCL: neuronal ceroid lipofuscinosis. ANCL: adult neuronal ceroid lipofuscinosis. JNCL: juvenile neuronal ceroid lipofuscinosis. INCL infantile neuronal ceroid lipofuscinosis. LINCL late-infantile neuronal ceroid lipofuscinosis

vJNCL: variant juvenile neuronal ceroid lipofuscinosis. cJNCL: lcassic juvenile neuronal ceroid lipofuscinosis; CBCL: Child Behavior Checklist. TRF: Teacher Report Form. CDI: Children’s Depression Inventory. SIB-R: Scales of Independent Behavior – Revised. YBOCS: Children’s Yale-Brown Obsessive Compulsive Scale. UBDRS: Unified Batten Disease Rating Scale. NPI-Q: Neuropsychiatric Inventory – Questionnaire. WISC-R: Wechsler Intelligence Scale for Children–Revised. PedsQL: Pediatric Quality of Life questionnaire. CGI-S: Clinical Global Impression scale’ Illness severity score. WCMC: Weill Cornell Medical College. WES Whole-exome sequencing. ADL: activities of daily living. NMS: neuroleptic malignant syndrome. SS: serotonin syndrome. OCD: obsessive–compulsive disorder. SARs: severe adverse reactions. Ris: risperidone. Cip: citalopram. ECT: electroconvulsive therapy. VPA: valproate. y: years. m: months. M: male. F: female. DSM-5: Diagnostic and Statistical Manual of Mental Disorders, Fifth Edition. MRI: magnetic resonance imaging. SPECT: single photon emission computed tomography. EEG: electroencephalography. CSF: cerebrospinal fluid. FDG-PET fluorodeoxyglucose position emission tomography

**Supplementary Table S2**. Standardized psychiatric and behavioral assessment tools used in NCL studies

| **Tool** | **Target age** | **Domains assessed** | **NCL subtype** | **References** |
| --- | --- | --- | --- | --- |
| CBCL | 6–18 y | Behavioral and emotional problems in children/adolescents (anxiety, depression, social problems, aggressiveness, etc.) | CLN3 | Bäckman et al.; 2005; Adams et al., 2006; Adams et al., 2010 |
| CDI | 7–17 y | Depressive symptoms in children and adolescents | CLN3 | Bäckman et al., 2005 |
| TRF | 6–18 y | Teacher assessment of behavioral and social problems | CLN3 | Bäckman et al., 2005 |
| SIB-R | 2 y–adult age | Adaptive skills and problematic behaviors | CLN3 | Adams et al., 2006 |
| YBOCS | 8–17 y | Obsessive–compulsive symptoms | CLN3 | Adams et al., 2006 |
| UBDRS | All ages | The behavioral scale of the UBDRS assesses eight symptom domains: sad mood, apathy, anxiety, aggression to self, aggression to others, stereotyped/repetitive behaviors, auditory hallucinations, and obsessions | CLN3 | Marshall et al., 2005; Adams et al., 2010; Cialone et al., 2012; Honingh et al., 2022 |
| NPI-Q | Adults (primarily ≥18 y) | Caregiver-rated assessment of 12 neuropsychiatric domains (delusions, hallucinations, agitation/aggression, depression/dysphoria, anxiety, euphoria/elation, apathy/indifference, disinhibition, irritability/lability, aberrant motor behavior, night-time disturbances, appetite/eating changes), also taking into account caregiver distress | CLN3 | Atee et al., 2024 |
| Bush–Francis Catatonia Rating Scale | ≥12 y | Three catatonic signs and symptoms across motor, behavioral, and affective domains (e.g., mutism, negativism, posturing, stereotypy, waxy flexibility, echolalia, echopraxia, withdrawal, excitement); includes both a screening instrument and a severity rating scale | CLN13 | Bayır et al., 2025 |

CBCL: Child Behavior Checklist. CDI: Children’s Depression Inventory. TRF: Teacher Report Form. SIB-R: Scales of Independent Behavior – Revised. YBOCS: Children’s Yale-Brown Obsessive Compulsive Scale. UBDRS: Unified Batten Disease Rating Scale. NPI-Q: Neuropsychiatric Inventory Questionnaire. y: years.

**Supplementary Table S3**. Psychotropic medications reported in the literature on NCL.

| **Author(s)** | **Population** | **Drug** | **Dosage** | **Indication (Symptoms/Diagnosis)** | **Effective?** | **Side effects** |
| --- | --- | --- | --- | --- | --- | --- |
| Bäckman et al., 2005 | 14 Finnish patients with JNCL, mean age 13.8 y (7–25) | Citalopram | 18.5 mg/day (5–30 mg) if >40 kg, or 0.5 mg/kg/day if <40 kg | Affective symptoms (depression, aggressiveness), anxiety | Good (2), satisfactory (6), fair (2) → mostly effective | Fatigue, weight gain, dizziness, agitation/uncontrollable behavior, possible extra-pyramidal symptoms |
|  |  | Risperidone | 2.8 mg/day (1–8.5) or 0.05 mg/kg/day | Psychotic symptoms | Good (2), satisfactory (2), fair (2) → moderately effective | Fatigue, weight gain, wakefulness |
|  |  | Olanzapine | 15 mg/day | Psychotic symptoms | Good (1), satisfactory (1), no response (1) → less effective | Fatigue, extra-pyramidal symptoms/aggravation |
|  |  | Quetiapine | 200 mg/day | Psychotic symptoms | Good (1) → effective in one severe case | Fatigue |
| Hinkebein et al., 1997 | 49-year-old female with ANCL (Kufs disease) | Phenothiazines (neuroleptics, initial treatment) | Not specified (short course during hospitalization) | Psychotic symptoms | Ineffective; worsened condition | Severe dystonic reaction: mutism, rigidity, abnormal posturing; became obtunded until drug stopped |
| Kulkarni, et al., 2021 | 39-year-old female, Kufs disease (*CTSF* mutation), resistant schizophrenia progressing to dementia | Aripiprazole, risperidone, amisulpride | Not specified | Psychotic symptoms (paranoid delusions, auditory hallucinations, withdrawn behavior) | Ineffective | None specifically reported |
|  |  | Clozapine | 200 mg/day | Treatment-resistant schizophrenia | Initially some improvement; then seizures appeared → discontinued | Seizures (frequent within 2 months, possible also after withdrawal) |
| Lee et al., 2010 | 20-year-old female with JNCL | Antidepressants, antipsychotics | Not specified | Depression, emotional lability, auditory hallucinations, paranoid delusions | Not specified | Not specified |
|  | 18-year-old male with JNCL | Risperidone | 0.5 mg twice daily | Behavioral dyscontrol: intermittent explosive tantrums, aggression, social inappropriateness | Not specified | Not specified |
| Reif et al., 2003 | 30-year-old woman with ANCL (Kufs disease, type B) | Mirtazapine + olanzapine (initially) | Not specified | Behavioral disturbances, psychotic symptoms (paranoia, agitation, delusions) | Partial improvement: cognitive decline continued | None reported |
|  |  | Fluphenazine (a typical oral neuroleptic) | Not specified | Catatonic psychosis (mutism, immobility alternating with agitation) | Ineffective; caused NMS | Severe: hyperthermia, rigidity, tremor, autonomic symptoms, high serum CK level |
|  |  | Diazepam, biperiden, dantrolene | Standard acute regimen | Treatment of NMS | Partial improvement | None significant |
|  |  | ECT | 10 sessions | Persistent catatonia after NMS | Gradual improvement of vegetative symptoms, rigidity, and mobility | None reported |
|  |  | Amantadine | Not specified | Post-NMS akinesia, mutism, cognitive slowing | Marked improvement in speech, movement, affect | None reported |
|  |  | quetiapine | Not specified | Recurrence of excitatory/fearful states after amantadine | Effective and well tolerated | None reported |
| Santavuori et al., 1993 | 42 Finnish JNCL patients (plus smaller INCL and LINCL groups) | Baclofen | 15–25 mg/day, up to 200 mg/day | Irritability, choreoathetotic behavior, late pain, motor restlessness (INCL and JNCL) | Very effective; reduced irritability and motor restlessness | None significant; intrathecal baclofen suggested in severe cases |
|  |  | Tizanidine | 1–9 mg/day, later 20–60 mg/day | Irritability, sleep dysfunction, motor hyperactivity | Effective, especially in combination with baclofen | Mild sedation |
|  |  | Levomepromazine (methotrimeprazine) | 1.25–2.5 mg 3–4×/day (<15 yrs); 6.25–30 mg/day (>15 y; up to 50 mg/day in psychosis) | Sleep disorder, psychotic/agitated states | Effective; improved sleep and reduced agitation | Sedation; higher doses used cautiously |
|  |  | Benzodiazepines (oxazepam, diazepam, midazolam) | Not specified (midazolam in severe sleep disorder) | Anxiety, restlessness, sleep disturbance, acute psychotic crises | Effective in acute situations | Sedation |
|  |  | Valproate | Not specified | Epilepsy, irritability, and sleep dysfunction | Effective in some children; abolished sleep disorder in two cases | Occasional sleep aggravation in others |
|  |  | Paraldehyde, chlorpromazine (IM) | As needed in acute psychotic crisis | Psychotic agitation (acute) | Effective temporarily | Not reported |
|  |  | Psychosocial & environmental interventions (rocking chair, bubble bath, water bed, reduced stimuli) | N/A | Anxiety, sleep disturbance, agitation | Effective in many patients when combined with pharmacologic therapy | None |
| Savvidou et al., 2022 | 4 adolescents (14–17 y) with CLN3 (homozygous or compound heterozygous 1.02 kb deletion in *CLN3*) | Sertraline + risperidone + olanzapine (Patient 1) | Sertraline 25 mg/day, olanzapine 5 mg/day | Anxiety and agitation | Ineffective; deterioration | Severe serotonin/NMS-like syndrome with hyperthermia (41°C), rigidity, rhabdomyolysis (CK > 3000 U/L), death |
|  | Male, 14–15 y (Patient 2) | Risperidone → clozapine | Risperidone 0.25 mg/day, clozapine 12.5 mg BID | Anxiety, panic attacks, hallucinations, agitation | Initial partial benefit; rapid worsening | Severe NMS-like syndrome; fever 41°C, CK 3964 U/L, rigidity, death |
|  | Female, 14.5 y (Patient 3) | Sertraline + olanzapine | Sertraline 50 mg/day; olanzapine 2.5–5 mg/day | Acute psychosis, hallucinations, apathy | Transient mild benefit | NMS; fever 42°C, CK 55 700 U/L, renal/liver failure, death |
|  | Female, 15 y (Patient 4) | Risperidone, olanzapine, haloperidol, quetiapine | Olanzapine 2.5–5 mg/day; quetiapine 25–75 mg/day; haloperidol 5 mg IM | Anxiety, hallucinations, agitation | Partial effect (quetiapine tolerated) | Multiple hyperthermia episodes; CK 20050 U/L; dystonia, rigidity; survived |
| Tobo et al., 1984 | Two Japanese siblings (male 49 y, female 51 y) with ANCL (Kufs disease); consanguineous family | ECT | 95 sessions (male); 19 sessions (female) | Episodic psychotic states misdiagnosed as schizophrenia (hallucinations, delusions, agitation, stupor) | Temporary slight improvement after some sessions; no lasting benefit | Cognitive decline; progressive stupor; no acute adverse effects described |
| Vercammen et al, 2003 | 23-year-old female with JNCL (*CLN3*: 1.02 kb del + 424delG) | Trazodone + oxazepam (prior to risperidone) | Not specified | Agitation, sleep problems | Mild benefit prior to NMS | Not reported |
|  |  | Risperidone | 0.5 mg/day | Hallucinations, agitation, inappropriate behavior | Not effective; caused severe NMS | Neuroleptic malignant syndrome: high fever (40.6°C), rigidity, tremor, oculogyric crises, elevated CK (16,822 U/L), autonomic instability; resolved after discontinuation |
|  |  | Diazepam + bromocriptine (after NMS onset) | Standard regimen | Treatment of NMS | Effective (fever abated in 2 days, serum CK normalized in 2 weeks) | None significant |
| Atee et al., 2024 | Female with Batten disease, blindness, dysarthria, seizures, irritability/lability | Olanzapine, mirtazapine | Not specified | Irritability/lability, aggression, anxiety, mood lability | Marked improvement of irritability and agitation with multimodal psychosocial interventions (pet therapy, assistive tech, sensory engagement, structured routine); NPI-Q distress score decreased from 11 to 8 | None reported |
| Özkara et al., 2017 | 2 siblings (female onset 18 y; male onset 26 y), consanguineous family, *CLN6* homozygous mutation (c.509A>G, p.Tyr170Cys) | Citalopram | Not specified | OCD and depression | Partial improvement of psychiatric symptoms | None reported |

NCL: neuronal ceroid lipofuscinosis. ANCL: adult neuronal ceroid lipofuscinosis. JNCL: juvenile neuronal ceroid lipofuscinosis. INCL: infantile neuronal ceroid lipofuscinosis. LINCL: late-infantile neuronal ceroid lipofuscinosis. NMS: neuroleptic malignant syndrome. ECT: electroconvulsive therapy. y: years. N/A: not available. OCD: obsessive–compulsive disorder. NPI-Q: NPI-Q: Neuropsychiatric Inventory Questionnaire. IM: intramuscular administration

**Supplementary Table S4**. Summary of reported psychotropic drug use and clinical responses in neuronal ceroid lipofuscinoses (NCLs).

| **Drug Class** | **Drug(s)** | **Reported Clinical Response** | **Reported Adverse Effects / Tolerability** | **Clinical Remarks** | **References** |
| --- | --- | --- | --- | --- | --- |
| Antidepressants (SSRIs, NaSSA) | Citalopram | Subjective improvement in affective symptoms, anxiety, and irritability in some JNCL patients | Fatigue, weight gain, mild dizziness; generally, well tolerated | Considered beneficial in mood and anxiety symptoms; commonly used at moderate doses (5–30 mg/day) | Bäckman et al., 2001; Özkara et al., 2017 |
|  | Sertraline | Limited or transient benefit; in several CLN3 adolescents, deterioration observed after co-administration with antipsychotics | Severe serotonin/NMS-like syndromes and fatal hyperthermia when combined with risperidone or olanzapine | Should be used with extreme caution; avoid combination with dopamine-blocking agents | Savvidou et al., 2022 |
|  | Mirtazapine | Reported improvement of anxiety, irritability, and sleep regulation in individual cases | Well tolerated; no significant side effects described | May help in behavioral lability and sleep disturbance; potential role in multimodal care | Reif et al., 2003; Atee et al., 2024 |
| Atypical antipsychotics | Risperidone | Variable clinical benefit in agitation or psychotic symptoms; partial response in some, no improvement in others | Reported extrapyramidal signs, sedation and weight gain. Neuroleptic malignant–like syndrome also reported | Reports highlight a risk of NMS-like episodes | Backman et al., 2001; Vercammen et al., 2003; Savvidou et al., 2022 |
|  | Olanzapine | Inconsistent clinical benefit; some transient improvement reported, but several cases of serious hyperthermic reactions | Fatigue, extrapyramidal symptoms, hyperthermia, and rhabdomyolysis | Reports highlight a risk of NMS-like episodes, particularly when combined with SSRIs | Bäckman et al., 2001; Savvidou et al., 2022 |
|  | Quetiapine | Subjective behavioral stabilization and reduction of agitation in isolated cases | Well tolerated; mild fatigue | Appears better tolerated than other antipsychotics; considered a relatively safer option if needed | Bäckman et al., 2001; Reif et al., 2003; Savvidou et al., 2022 |
|  | Clozapine | Initial behavioral improvement followed by relapse and seizure onset in single reports | Seizures and intolerance even after withdrawal | Use limited to refractory psychotic states; requires EEG monitoring | Kulkarni et al., 2021 |
| Typical antipsychotics | Phenothiazines, fluphenazine, haloperidol, chlorpromazine | No sustained benefit; often clinical worsening (catatonia, rigidity, mutism) | Severe extrapyramidal and autonomic reactions, including NMS and dystonic crises | Not recommended as first choice in NCL due to patients’ extreme susceptibility to motor and autonomic side effects | Hinkebein et al., 1997; Reif et al., 2003; Savvidou et al., 2022 |
| Muscle relaxants / Anxiolytics | Baclofen | Reported reduction of irritability, motor restlessness, and choreoathetosis (especially in JNCL/INCL) | Mild sedation only; generally safe | Consistently beneficial for motor and affective hyperexcitability | Santavuori et al., 1993 |
|  | Tizanidine | Improvement of sleep and behavioral regulation, often used in combination with baclofen | Mild dose-related sedation | Useful adjunct to baclofen in managing irritability and sleep–wake disturbances | Santavuori et al., 1993 |
|  | Benzodiazepines (oxazepam, diazepam, midazolam) | Reported calming and sedative effect in acute agitation, anxiety, and sleep problems | Sedation; potential tolerance with chronic use | Suitable for short-term management of acute behavioral crises | Santavuori et al., 1993; Reif et al., 2003 |
| Mood stabilizers / others | Valproate | Benefit described for epilepsy and irritability; inconsistent for sleep regulation | Hyperammonemia, risk factor for rapid exacerbation of complex movement disorder | May aid behavioral stabilization in epileptic patients | Santavuori et al., 1993; Larsen et al., 2014; Johannsen et al., 2016 |
| Non-pharmacological interventions | Psychosocial and sensory therapies (structured environment, pet therapy, sensory engagement) | Reported reduction of agitation, anxiety, and caregiver distress; improved daily rhythm | No adverse effects | Core component of multimodal management; may allow lower psychotropic doses | Santavuori et al., 1993; Atee et al., 2024 |

NCL: neuronal ceroid lipofuscinosis. INCL: infantile neuronal ceroid lipofuscinosis. JNCL: juvenile neuronal ceroid lipofuscinosis. NMS: neuroleptic malignant syndrome. SSRIs: selective serotonin reuptake inhibitors.
